# Discovery of Molecular Markers to Discriminate Corneal Endothelial Cells in the Human Body

**DOI:** 10.1371/journal.pone.0117581

**Published:** 2015-03-25

**Authors:** Masahito Yoshihara, Hiroko Ohmiya, Susumu Hara, Satoshi Kawasaki, FANTOM consortium, Yoshihide Hayashizaki, Masayoshi Itoh, Hideya Kawaji, Motokazu Tsujikawa, Kohji Nishida

**Affiliations:** 1 Department of Ophthalmology, Osaka University Graduate School of Medicine, Suita, Osaka, Japan; 2 Division of Genomic Technologies, RIKEN Center for Life Science Technologies, Yokohama, Kanagawa, Japan; 3 Preventive Medicine and Applied Genomics Unit, RIKEN Advanced Center for Computing and Communication, Yokohama, Kanagawa, Japan; 4 RIKEN Preventive Medicine and Diagnosis Innovation Program, Wako, Saitama, Japan; Instituto Butantan, BRAZIL

## Abstract

The corneal endothelium is a monolayer of hexagonal corneal endothelial cells (CECs) on the inner surface of the cornea. CECs are critical in maintaining corneal transparency through their barrier and pump functions. CECs *in vivo* have a limited capacity in proliferation, and loss of a significant number of CECs results in corneal edema called bullous keratopathy which can lead to severe visual loss. Corneal transplantation is the most effective method to treat corneal endothelial dysfunction, where it suffers from donor shortage. Therefore, regeneration of CECs from other cell types attracts increasing interests, and specific markers of CECs are crucial to identify actual CECs. However, the currently used markers are far from satisfactory because of their non-specific expression in other cell types. Here, we explored molecular markers to discriminate CECs from other cell types in the human body by integrating the published RNA-seq data of CECs and the FANTOM5 atlas representing diverse range of cell types based on expression patterns. We identified five genes, *CLRN1*, *MRGPRX3*, *HTR1D*, *GRIP1* and *ZP4* as novel markers of CECs, and the specificities of these genes were successfully confirmed by independent experiments at both the RNA and protein levels. Notably none of them have been documented in the context of CEC function. These markers could be useful for the purification of actual CECs, and also available for the evaluation of the products derived from other cell types. Our results demonstrate an effective approach to identify molecular markers for CECs and open the door for the regeneration of CECs *in vitro*.

## Introduction

Cornea is a transparent tissue located at the front of the eye and it serves as the main refractive element of the eye. It consists of three layers; epithelium, stroma, and endothelium. The corneal epithelium covers the front of cornea and acts as a barrier. The stroma is the thickest layer of the cornea and provides the cornea structural strength. Finally, the corneal endothelium is a monolayer of hexagonal cells on the inner surface of the cornea, attached to its basement membrane termed Descemet’s membrane. Cells consisting of endothelial layers (corneal endothelial cells, CECs) play an essential role in maintaining the corneal transparency by their barrier and pump function through Na^+^-K^+^-ATPase and bicarbonate-dependent pump that regulates the hydration between the stroma and the anterior chamber[1,2].

CECs *in vivo* have a limited capacity in proliferation since they are arrested at the G1-phase of the cell cycle[3,4]. Loss of a small number of CECs *in vivo* can be compensated by migration and enlargement of remaining CECs. However, loss of a significant number of CECs caused by injury[5,6] or inherited diseases[7] cannot be compensated. The loss of CECs decreases function of the pump[8], which results in serious edema of cornea called bullous keratopathy and severe visual loss.

The most effective way to treat these serious disorders in clinical settings at this moment is corneal transplantation, where endothelial failure is one of the most common indications[9]. However, corneal transplantation suffers from global shortage of donation with healthy conditions[10]. To overcome the shortage, a large number of studies are being made in the field of tissue bioengineering to generate alternative CECs from different kind of sources – *ex vivo* expanded CECs[11], or multipotent cells such as neural crest-derived stem cells from iris[12] or corneal stroma[13] and embryonic stem cells[14].

Because cultured CECs have a limited proliferative and passaging ability[15], induction of CECs from stem cells seems to be more feasible. These induced CECs are a mixture of different cell types, which may include poorly characterized or unknown cellular states. It is therefore crucial to discriminate actual CECs from other undesired cells. At present, a few proteins such as ZO-1[16], Na^+^-K^+^-ATPase[17] and N-cadherin[18], are used as CEC markers, however, they are also expressed in other cell types[19–21] (S1 Table). Isolation of CECs based on these markers is far from satisfactory, considering that original stem cells have the ability to differentiate into a variety of cell types. Although substantial efforts to find specific markers of CECs have been made so far, it remains incomplete for a long time because of the limited proliferative ability of CECs both *in vivo* and *in vitro* and the small number of cells existing *in vivo*.

Recent developments in next-generation sequencing technology has made it possible to identify and quantify expressed RNA species across the genome even from small amount of samples[22]. A few studies tackled genome-wide RNA profiling of CECs[23–25], however, their expression analyses compared only a limited number of tissue types including CECs. Given heterogeneity of cellular states induced to CECs *in vitro*, a broader survey of different cell types is crucial to identify molecular markers specific only to CECs.

In this study, we tackled a problem to identify molecular markers to discriminate CECs in the human body. Given approximately 400 human cell types reported so far[26], identification of specific transcripts requires examination of their expressions in a large panel of samples. We approached this step by using the Functional Annotation of Mammalian Genome 5 (FANTOM5) expression atlas[27], consisting of 975 human samples including primary cells, tissues and cancer cell lines. We started the computational screening from a set of published transcriptome data on CECs[23] since the FANTOM5 expression atlas does not include CEC profiles, and we subsequently excluded transcripts expressed in a broad range of samples. We followed up the resulting marker candidates by experimental validation at the level of RNA by using quantitative reverse transcription PCR (qRT-PCR) as well as protein by immunofluorescence staining. This work is part of the FANTOM5 project. Data downloads, genomic tools and co-published manuscripts are summarized here http://fantom.gsc.riken.jp/5/.

## Materials and Methods

### 1. Bioinformatics analysis

#### 1–1. RNA-seq data obtained from CECs

RNA-seq is a method to obtain transcriptome profiles by sequencing random fragment of long RNAs[28], and Chen et al. studied CECs with RNA-seq[23]. This dataset GSE41616, downloaded from the Gene Expression Omnibus (GEO) database[29], consists of three donations obtained from adult CECs (31, 56 and 64 years old) and two donations obtained from fetal CECs (16–18 weeks of gestation). The RNA-seq reads were aligned to the human reference genome (hg19) by using TopHat (version 1.4.1), and the results were used to assemble transcript models by Cufflinks package (version 2.1.1)[30] where Gencode v14 was used as reference transcripts. Cuffmerge was used to merge the transcripts of each of the adult and fetal datasets. Cufflinks was used to quantify gene expression values as Fragments Per Kilobase of exon per Million mapped fragments (FPKM). The resulting transcripts were associated with genes based on the gencode transcripts.

#### 1–2. CAGE data, obtained from samples across the human body

CAGE (Cap Analysis Gene Expression) is a method to obtain transcriptome profiles by sequencing 5’-ends of capped RNAs, which identifies transcription starting sites (TSSs) and quantifies their activities. The FANTOM5 project surveyed TSSs in a large collection of primary cells, tissues, and cell lines, which consists of 975 human samples including more than 180 cell types[27]. On the human reference genome, ∼180,000 regions are identified as peaks of the TSS signals and associated with transcripts and genes based on their genomic coordinates. Activities (or expressions) of the TSS peaks are quantified as TPM (tags per million) across the 975 samples based on the CAGE reads obtained. We downloaded the data set from the web site (http://fantom.gsc.riken.jp/5/) and used in the following analyses.

#### 1–3. Selection of the candidate markers specific to CECs from the RNA-seq and the FANTOM5 CAGE data

**Fig 1:**
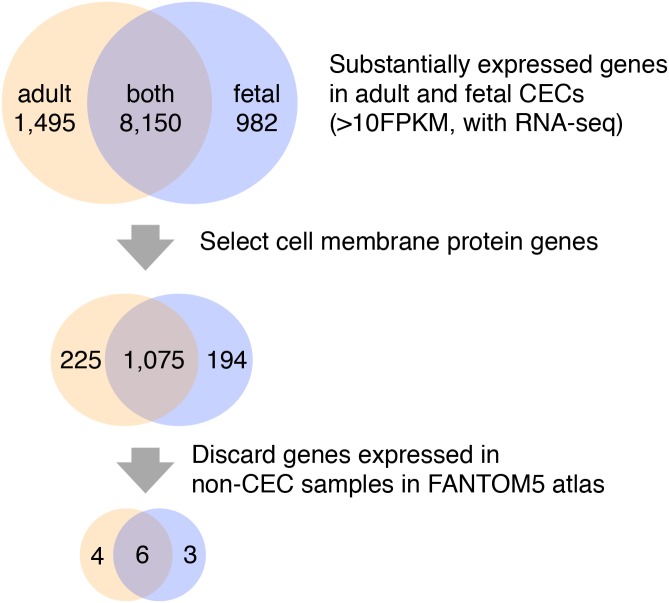
Bioinformatics analysis flow chart FPKM: Fragments Per Kilobase of exon per Million mapped fragments.

As shown in Fig. 1, we started the analysis from selection of genes that express more than 10 FPKM in either the adult or the fetal CEC RNA-seq data. We narrowed down the list of genes by selecting only annotated as cell membrane protein by using GO terms[31] (Table 1). We further narrowed down the gene list by discarding ones that expressed more than 10 TPM in over 5 normal tissues or primary cells based on the FANTOM5 CAGE data (Fig. 1).

**Table 1:** Cell membrane protein coding genes which express more than 10 FPKM in either or both of adult and fetal corneal endothelial cells.

**GO term**	**GO ID**	**Both**	**Adult**	**Fetal**
plasma membrane	GO:0005886	762	155	124
integral to plasma membrane	GO:0005887	215	49	57
cell surface	GO:0009986	111	20	28
apical plasma membrane	GO:0016324	59	18	9
basolateral plasma membrane	GO:0016323	50	12	8
external side of plasma membrane	GO:0009897	31	9	9
lateral plasma membrane	GO:0016328	10	0	4
basal plasma membrane	GO:0009925	8	2	2
extrinsic to plasma membrane	GO:0019897	8	2	0
apicolateral plasma membrane	GO:0016327	5	0	1
anchored to external side of plasma membrane	GO:0031362	4	0	0
anchored to plasma membrane	GO:0046658	2	2	0
extrinsic to external side of plasma membrane	GO:0031232	2	0	0
intrinsic to external side of plasma membrane	GO:0031233	1	1	0
intrinsic to plasma membrane	GO:0031226	1	1	0
cell outer membrane	GO:0009279	1	0	0
external side of cell outer membrane	GO:0031240	0	0	0
integral to cell outer membrane	GO:0045203	0	0	0
**Total**		**1,075**	**225**	**194**

Both: genes substantially expressed both in adult and fetal corneal endothelial cells.

Adult: genes substantially expressed only in adult corneal endothelial cells.

Fetal: genes substantially expressed only in fetal corneal endothelial cells.

### 2. Human Ocular Tissue Preparation

All *human samples were handled according* to the tenets of the *Declaration of Helsinki*. Research-grade corneoscleral rims and whole eyeballs from cadaver human donors considered unsuitable for transplantation were procured from Sight Life (Seattle, WA).

#### 2–1. RNA preparation from human corneal endothelium

A donor cornea was preserved and transported in Optisol-GS (Bausch & Lomb, Rochester, NY) at 4°C, and was used within four days from preservation. The age of donor was 58 years old.

The corneoscleral rim was washed with phosphate-buffered saline (PBS) three times, then it was placed under the dissecting microscope (SZ61; Olympus, Tokyo, Japan) with the endothelium side up in a Petri dish, and the endothelium and Descemet's membrane were carefully dissected from the cornea along Schwalbe's line. The Descemet's membrane with its attached corneal endothelium was rapidly transferred into RNA later RNA Stabilization Reagent (QIAGEN Inc., Valencia, CA). Total RNA was extracted by Qiagen miRNeasy Mini Kit (QIAGEN Inc.) according to the manufacturer's protocol.

#### 2–2. RNA preparation from human ocular and non-ocular tissues

To prepare RNA extracts from human ocular tissues, four whole eyeballs from two donors were preserved and transported in moist chamber at 4°C, and were used within five days from preservation. The ages of donors were 75 and 79 years old.

The whole globes were first divided into the anterior segments and the posterior cups. Each tissue was carefully isolated using sterile forceps. After the endothelium and Descemet's membrane were peeled, the corneoscleral rims were punched out by using an 8.0-mm diameter trephine. Central corneal parts and limbal parts were treated with Dispase I (Godo Shusei, Tokyo) overnight at 4°C, and corneal epithelium and limbal epithelium were separated from stroma. All the isolated tissues were rapidly transferred into Isogen RNA extraction reagent (Nippon Gene, Tokyo). Total RNA was extracted using the Isogen RNA extraction kit according to the manufacturer's protocol.

*The RNA* samples from non-ocular primary tissues were purchased (*Human total RNA master panel* II #*636643*; *Clontech*, Mountain View, CA). In addition to this panel, Human Kidney Total RNA (#AM7976; Ambion, Austin, TX) and Human Pancreas Total RNA (#AM7954; Ambion) were also purchased.

#### 2–3. RNA preparation from human cultured CECs

To prepare samples for CEC culture, four donor corneas were preserved and transported in Optisol-GS at 4°C, and were used within 6 days from preservation. The ages of donors ranged from 14–25 years. Descemet's membrane with corneal endothelium was isolated as mentioned above. The isolated tissue was incubated for 1 hour at 37°C in a medium containing Dulbecco’s modified Eagle’s medium (DMEM; Invitrogen), 1.2 U/mL dispase II (Godo Shusei) and 1% Antibiotic-Antimycotic (Anti-Anti; Invitrogen/Gibco). Hereby, CECs were separated from Descemet’s membrane. After gentle centrifugation, the cells were suspended in culture medium containing DMEM, 50 U/mL penicillin, 50 μg/mL streptomycin, 10% fetal bovine serum (ICN Biomedicals, Inc., Aurora, OH), and 2 ng/mL basic fibroblast growth factor (bFGF; invitrogen). The cells were incubated on dishes coated with cell attachment reagent (FNC coating mix; Athena ES, Baltimore, MD) in an incubator at 37°C with humidified atmosphere of 10% CO_2_. Total RNA was extracted using the Isogen RNA extraction kit. All the cells used for RNA extraction were harvested at full confluence during the first passage.

#### 2–4. Quantitative reverse transcription polymerase chain reaction (qRT-PCR)

Total RNAs were inputted into reverse transcription-polymerase chain reaction with the SuperScript III First-Strand Synthesis System (Invitrogen, Carlsbad, CA), and cDNA was used as a template for quantitative PCR. Quantitative PCR was performed using the ABI Prism 7500 Fast Sequence Detection System (Applied Biosystems, Foster City, CA). SYBR Pre-mix Dimer Eraser (Takara, Shiga, Japan) was used, and expression values were normalized to the housekeeping gene β-actin *(ACTB)* as an internal control. The thermocycling program was performed as follows: an initial cycle at 95°C for 30 sec, followed by 45 cycles of 95°C for 5 sec, 60°C for 30 sec and 72°C for 30 sec. Data were obtained from duplicate experiments, and Ct values above 40 were regarded as not expressed.

The list of primers used in this study is indicated in Table 2.

**Table 2:** The sequences of the primers used in the study.

**Gene**		**Primer Sequence**	**Product Size (bp)**	**Accession Number**
ACTB	Forward	5’-ACAGAGCCTCGCCTTTGC-3’	75	NM_001101
	Reverse	5’-GCGGCGATATCATCATCC-3’		
NSF	Forward	5’-CCTATTGGCCCTCGATTTTC-3’	106	NM_006178
	Reverse	5’-GGCTAGTGGTCCCAATGATAAG-3’		
PKD1	Forward	5’-AAGACACCCACATGGAAACG-3’	72	NM_001009944
	Reverse	5’-CCAGCGTCTCTGTCTTCTCC-3’		
SCNN1D	Forward	5’-TGGAGCTGCTACACAACACC-3’	82	NM_001130413
	Reverse	5’-GAGCAGGTCTCCACCATCAG-3’		
CNTN3	Forward	5’-CCATGGAAACAGTTGATCCTG-3’	96	NM_020872
	Reverse	5’-GCTGTTGCTGGGTTCTTTG-3’		
CNTN6	Forward	5’-TTCTGAGTCGGAAGGCAAAG-3’	79	NM_014461
	Reverse	5’-CGGACAGATACTGTGCTTCTTG-3’		
PCDHB7	Forward	5’-ATTTTGTGCGGTCGCTCTAC-3’	106	NM_018940
	Reverse	5’-TCCCCATTACTTCCGGTATC-3’		
PPIP5K1	Forward	5’-CTTTCCCTACGTCAAGTGAGTG-3’	105	NM_014659
	Reverse	5’-GCTGCTGTGCATGGAATC-3’		
CLRN1	Forward	5’-AATGCAGTACGGGCTTTTCC-3’	109	NM_174878
	Reverse	5’-GCTCACTGGGATTGCTTTG-3’		
MRGPRX3	Forward	5’-GGAGGTCTTCACCACTGGAC-3’	90	NM_054031
	Reverse	5’-ACCCAAGACTGGGATGGTTG-3’		
GLP1R	Forward	5’-GCAGAAATGGCGAGAATACC-3’	97	NM_002062
	Reverse	5’-TTCATCGAAGGTCCGGTTG-3’		
HTR1D	Forward	5’-CATGCGTTTCTTCCACTGAG-3’	85	NM_000864
	Reverse	5’-CATCGGCACTGCAAATACTG-3’		
GRIP1	Forward	5’-ATGTGGACAAGAAGCAGCAC-3’	102	NM_021150
	Reverse	5’-GGAGTTTTGGCAACTTCGAC-3’		
ZP4	Forward	5’-AAACAGGCCCTCAGGGGA-3’	88	NM_021186
	Reverse	5’-GACAGGTCACCACACAGGAT-3’		

#### 2–5. Immunofluorescence staining

A donor cornea was preserved and transported in Optisol-GS at 4°C, and was used within 11 days from preservation. The age of donor was 27 years old.

Fresh corneal tissues were embedded in optimal cutting temperature (OCT) compound and frozen sections were cut using a microtome-cryostat (HM560, Thermo Fisher Scientific Inc., Walldorf, Germany) in 10 μm. After drying for 30 minutes at room temperature, tissue sections were washed with Tris-buffered saline (TBS; Takara) 3 times, and incubated with TBS containing 5% donkey serum and 0.3% Triton X-100 for 1 hour to block non-specific reactions. Each section was then incubated with primary antibodies listed in Table 3 at 4°C overnight. Subsequently, slides were again washed with TBS 3 times, and incubated with a 1: 200 dilution of their respective Alexa Fluor 488-conjugated secondary antibodies (Life Technologies) and 2μg/mL Hoechst 33342 (#B2261, Sigma-Aldrich) for 2 hours at room temperature. The slides were mounted with a drop of Permafluor mountant (Thermo Scientific) to reduce photobleaching and observed by fluorescent microscopy (Axio Observer D1; Carl Zeiss Jena Gmbh, Jena Germany). For each of the primary antibodies, isotype specific rabbit IgG and goat IgG (#AB-105-C, #AB-108-C; R&D Systems, Minneapolis, MN) were used as negative controls at the same dilution as the primary antibodies.

**Table 3:** List of primary antibodies.

**Name**	**Company**	**Species and Type**	**Dilution Used**
**CLRN1 clarin 1 Cat No. sc-69073**	Santa Cruz Biotechnology, Inc. Santa Cruz, CA.	Goat pAb	1:50
**MRGPRX3 MAS-related GPR, member X3 Cat No. ab140863**	Abcam, Cambridge, MA	Rabbit pAb	1:25
**HTR1D 5-hydroxytryptamine receptor 1DCat No. ab140486**	Abcam, Cambridge, MA	Rabbit pAb	1:150
**GRIP1 glutamate receptor interacting protein 1 Cat No. ab122514**	Abcam, Cambridge, MA	Rabbit pAb	1:100
**ZP4 zona pellucida glycoprotein 4 Cat No. LS-C160968**	LifeSpan BioSciences, Inc. Seattle,WA	Rabbit pAb	1:50

## Results

### CEC specific marker candidates identified by bioinformatic analysis

We started our analyses from a collection of publicly available transcriptome data. As a resource of expressed genes in CECs *in vivo*, we obtained RNA-seq data produced by Chen et al.[23], which includes three adult CECs (31, 56 and 64 years old) and two fetal CECs (16–18 weeks of gestation). We examined two corneal epithelial cell specific markers, *KRT3* and *KRT12*[32,33] to confirm the data integrity as CECs, and we found that the corneal epithelial cell markers are abundant in one adult profile (the 56 year old donor) of the five CEC profiles. Since epithelial cells are likely contaminated within the profile, only the remaining four transcriptome profiles were used in the following analyses. The fetal profiles were included to recover genes expressed in CECs even if they were weakly expressed in the adult samples. As a resource of gene expressions representing a wide coverage of the human body, we used the FANTOM5 expression atlas[27] that consists of TSS activities across the genome in 975 samples, quantified by using a single molecule sequencer[34,35]. The atlas includes more than five hundred samples obtained from human primary cells, representing almost two hundred unique cell types, but it does not contain any samples from CECs. We used the atlas as a background set, i.e. to exclude genes expressing in other cell types or tissues.

We performed computational screening of candidate markers as shown in Fig. 1, which consists of the following three steps: 1) Selection of substantially expressed genes in CECs, 2) selection of membrane proteins, and 3) exclusion of genes expressed in other cells or tissues. We selected only genes annotated as membrane proteins in the step 2) so that we could use the marker genes to isolate cells by cell sorter in future. As a result of 1), we obtained 10,627 genes substantially expressed in CECs. We found that 1,495 genes are present only in adult CECs, 982 genes only in fetal CECs, and 8,150 genes in both of the adult and fetal CECs. Next we narrowed them down by selection of membrane protein and found 225, 194, 1,075 genes respectively (Fig. 1). Finally we excluded genes if their TSSs are active in more than 5 normal tissues or primary cells. As a result, we found 13 candidate markers consisting of four genes (*PPIP5K1*, *CLRN1*, *MRGPRX3*, *GLP1R*) in adult CECs, three genes (*CNTN3*, *PCDHB7*, *HTR1D*) in fetal CECs, and six genes (*GRIP1*, *NSF*, *PKD1*, *SCNN1D*, *ZP4*, *CNTN6*) in both of the adult and fetal CECs (Table 4).

**Table 4:** The expression levels of 13 CEC marker candidates in the RNA-seq data and the FANTOM5 database.

**Gene**	**RNA-seq FPKM value**		**FANTOM5 CAGE**	
	**adult CEC**	**fetal CEC**	**Primary sample expresses highest (tpm)**	**Primary samples express >10tpm**
**Substantially expressed only in adult CECs**				
PPIP5K1	17.39	6.17	None	0
CLRN1	14.15	0.56	lens epithelial cells(22.91)	2
MRGPRX3	11.16	0.26	Malassez-derived cells(26.14)	1
GLP1R	10.85	2.64	fetal heart (10.51)	1
**Substantially expressed only in fetal CECs**				
CNTN3	5.36	19.86	None	0
PCDHB7	1.11	11.14	dura mater(9.37)	0
HTR1D	7.41	10.48	small intestine(12)	2
**Substantially expressed both in adult and fetal CECs**				
GRIP1	39.33	22.70	fetal temporal lobe(6.46)	0
NSF	31.58	14.09	pineal gland(7.84)	0
PKD1	24.99	38.47	aorta(8.45)	0
SCNN1D	21.72	28.05	granulocyte macrophage progenitor(22.21)	4
ZP4	12.22	56.52	None	0
CNTN6	11.48	22.09	cerebellum(24.20)	4

### Expression specificities across the whole body and ocular tissues at the level of RNA

To validate the 13 candidate markers at the level of RNA, we designed experiments independently to the computational screening. At first, we performed qRT-PCR to examine their expression levels between adult human CECs *in vivo* and other non-ocular 22 primary tissues (S1 Fig). We found that expressions of all the examined genes are regulated tissue-dependent manner, which is consistent with the computational screening above. We also found that all of the examined genes do not show ideal expression patterns as CEC markers necessarily. For example, the expression levels of *NSF*, *PKD1*, *SCNN1D*, *CNTN3*, *CNTN6*, and *PCDHB7* in CECs were lower than their expression in some of the other samples examined. *PPIP5K1* and *GLP1R* exhibited the highest expression level in CECs across the samples but were detected in some of the other examined samples. By exclusion of these eight genes, we narrowed down the candidate genes into five genes, *CLRN1*, *MRGPRX3*, *HTR1D*, *GRIP1*, and *ZP4*.

**Fig 2:**
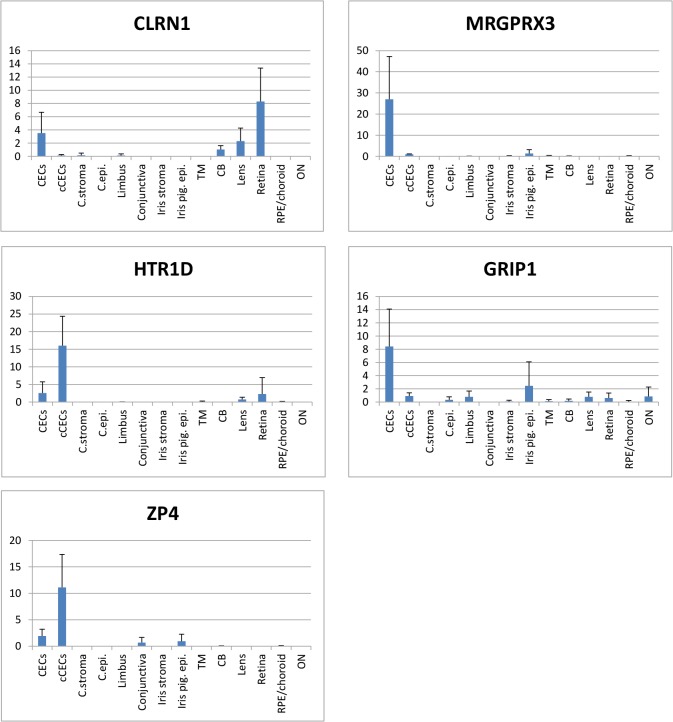
qRT-PCR analysis of 6 corneal endothelial cell marker candidate genes within ocular tissues. CECs: corneal endothelial cells, cCECs: cultured corneal endothelial cells, C.stroma: corneal stroma, C.epi.: corneal epithelial cells, iris pig. epi.: iris pigment epithelial cells, TM: trabecular meshwork, CB: ciliary body, RPE: Retinal pigment epithelial cells, ON: optic nerve. Y axis indicates % ACTB, and error bars represent standard deviation of four replicates.

Next, we examined expression levels of the five candidates in the ocular tissues and cultured CECs by qRT-PCR analysis, where four eyes from two independent donors were used for the ocular tissue analysis (Fig. 2). We successfully confirmed the presence of these five genes in CECs and their tissue-specific expressions, while they were also found in a few ocular tissues mostly at lower levels. For example, *CLRN1* was present in ciliary body, lens and retina, *HTR1D* in retina, *ZP4* in conjunctiva and iris pigment epithelial cells. Considering that corneal stroma is adjacent to CECs and it originates from cranial neural crest as CECs, absence or lower expression at corneal stroma is one of the important features to be useful as CEC marker. *CLRN1* was expressed at quite lower level in corneal stroma than in CECs, and the remaining four genes were not expressed in corneal stroma. Our results demonstrated their specific expression patterns across the human body and their absence or limited amount of expression levels in corneal stroma, suggesting that monitoring RNA expression levels of all the five genes is effective to discriminate CECs from the other cells.

### Exclusive staining of CECs within corneal tissue

**Fig 3:**
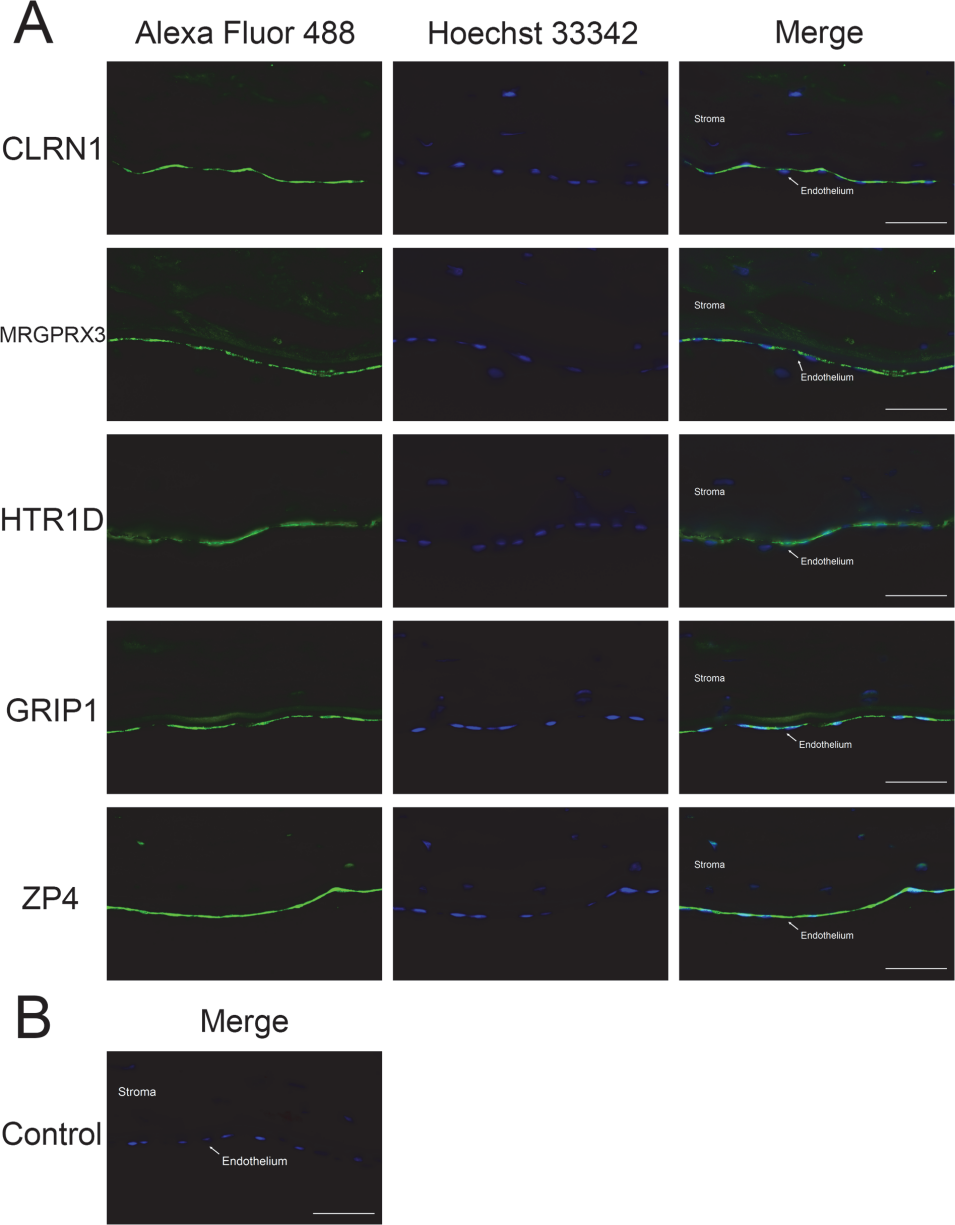
Immunofluorescence staining of human donor corneal tissue sections. (A) Green signals represent the expression of each protein detected by the specific antibody. (B) Negative control by using isotype specific rabbit IgG (green signal) and goat IgG (red signal) as primary antibodies. Hoechst 33342-stained nuclei are shown in blue. A white arrow indicates the corneal endothelium. All scale bars indicate 50 μm.

Finally, to confirm the expression of these five markers in protein level, we examined their expressions by immunofluorescence staining of human donor corneal tissue sections (Fig. 3) using the antibodies listed in Table 3. Remarkably all these staining indicate corneal endothelium. Interestingly, the staining patterns were different depending on the antibody. Anti-HTR1D and -ZP4 antibodies stained corneal endothelium exclusively. Although the expression level of *ZP4* in CECs was very low in qRT-PCR analysis, its antibody intensely stained corneal endothelium. It may suggest rapid degradation of mRNAs in contrast to sustained proteins on the cell surface. Anti-CLRN1 antibody staining showed slight signal from corneal stroma, which is consistent with the qRT-PCR experiments above, however, we could still discriminate CECs from stroma only by this staining pattern. Anti-MRGPRX3 antibody stained corneal endothelium and stroma, but did not stain Descemet’s membrane, and anti-GRIP1 antibody stained not only corneal endothelium but also Descemet’s membrane. We confirmed the utilities of the five genes as CEC markers at protein level, as well as the RNA level. Any single protein of the five markers would be useful to specify CECs *in vivo*, and their combinatorial use should be quite strict in discriminating CECs from a variety of cells.

## Discussion

In this study, we discovered novel CEC specific markers, CLRN1, MRGPRX3, HTR1D, GRIP1 and ZP4, that enabled us to discriminate CECs from other cells in the human body. We started the marker exploration from computational screening. Of the thirteen candidates identified, five genes were confirmed by the subsequent experiments (5/13; 38% success rate). Our approach based on careful curation of CEC transcriptome data and the use of wide coverage of expression atlas generated by FANTOM5 as a background set enabled us to identify novel CEC marker candidates which rarely express other than CECs. Although the set of FANTOM5 transcriptome profiles represent the largest number of cell types assayed in a single project, there remain missing cell types and we cannot reject possibilities that the identified candidates may be active in those cells. Our follow-up experiments based on qRT-PCR for CECs and several tissues enabled us to select five markers by excluding eight genes active in the tissues, which demonstrate both of the power and the limitation in our approach using computational screening. Since the qRT-PCR experiments in S1 Fig took tissues consisting of a variety of cell types, we performed additional qRT-PCR experiments for ocular tissues, each of which consists of limited types of cells. We also performed immunofluorescence staining for corneal tissue, and confirmed precise patterns of the five marker expressions. The series of analyses demonstrated the utilities of the selected genes as CEC molecular markers.

To our knowledge, this is the first report of the presence of these proteins in CECs *in vivo*. CLRN1 is known as the causative gene product of Usher syndrome type IIIa which causes deafness and visual impairment phenotypically similar to retinitis pigmentosa[36]. Its expression has been reported in glial cells in the retina and cochlear hair cells of the inner ear, but its molecular function remains unknown[37]. MRGPRX3 is considered to be involved in sensory neuron regulation and in the modulation of pain[38,39], HTR1D is a subtype of serotonin receptors, and distributed in nerve fibers[40], GRIP1 is a member of the glutamine receptor interacting protein family and enriched in synaptic plasma membrane [41], and ZP4 is an extracellular matrix surrounding the oocyte[42] in which it plays an important role in inducing acrosome reaction[43]. Most of them have been reported to be expressed in nervous tissues, which supports that CECs are derived from neural crest cells. However, their functions and roles in CECs remain to be elucidated.

Based on the qRT-PCR results shown in S1 Fig and Fig. 2, *MRGPRX3* is highly and exclusively expressed in CECs, and this gene should be the first choice as a molecular marker to discriminate CECs from other cell types. It is noteworthy that *MRGPRX3* is completely absent in corneal stroma which is adjacent to CECs and important to be discriminated because corneal stroma also originates from cranial neural crest as is the case for CECs. Interestingly, it is expressed at quite low level in heart, salivary gland and thymus that are all derived from neural crests.

The FANTOM5 expression atlas and our qRT-PCR analysis indicate that *ZP4* is evidently expressed only in CECs and not expressed in any other non-ocular tissues. Notably, the expression level of *ZP4* in cultured CECs is higher than CECs *in vivo*. Similarly, expression level of *HTR1D* was also higher in cultured CECs than CECs *in vivo*. Cultured CECs are essential for the investigation of physiology and pathology of CECs, and these two markers should be more useful as markers of cultured CECs than other candidates. Intriguingly, furthermore, the RNA-seq data showed that these two are expressed more in fetal CECs than in adult CECs, which suggests that they are more highly expressed in undifferentiated state of CECs. These data indicate that *ZP4* and *HTR1D* should be ideal markers for the discrimination of actual CECs during differentiation induced from multipotent stem cells.

Our analysis also indicated that *CLRN1* was rarely expressed in other non-ocular tissues. Although *CLRN1* was expressed in some other ocular tissues at RNA level, immunofluorescence staining analysis demonstrated its distinct expression pattern to CECs which enabled us to distinguish CECs from corneal stroma by itself. On the other hand, the remaining four genes were completely absent in corneal stroma according to the qRT-PCR analysis.

Previously, several marker candidates of CECs have been reported. Recently, Chng et al.[24] reported that *SLC4A11*, *COL8A2* and *CYYR1* could be useful to identify CECs. Cheong et al.[25] aimed to discover cell surface markers of CECs and identified *GPC4* and *CD200*. They analyzed the gene expression of CECs and corneal stroma by using RNA-seq, and identified genes that were highly expressed in CECs but lowly or not expressed in corneal stroma. Indeed, these markers could be useful to distinguish CECs from corneal stroma, but they were expressed in many other tissues according to the FANTOM5 expression atlas as shown in S1 Table. In our study, therefore, these markers were excluded at the stage of computational analysis. Compared with these previously reported markers, our five markers presented here show more strict specificity to CECs and might be more useful for the discrimination of CECs.

In conclusion, we identified five genes, *CLRN1*, *MRGPRX3*, *HTR1D*, *GRIP1* and *ZP4* as novel markers to discriminate CECs at the RNA and protein levels. Our results demonstrated their expression specificities, which would be applicable as CEC marker in particular for cell cultures consisting of unknown or mixtures of cell types. This is also the first report to examine the expression of these genes *in vivo* ocular tissues in depth, which may provide a clue to novel aspects of CEC functions and its transcriptional states. Given that the approach taken here is not very specific to CECs necessarily, our study sheds possibilities to explore novel molecular markers specific in other cell types, which is becoming highly important in clinical research.

## Supporting Information

S1 Fig qRT-PCR analysis of 13 corneal endothelial cell marker candidate genes across the whole body. CECs: corneal endothelial cells, A-brain: adult brain, F-brain: fetal brain, SC: spinal cord, SG: salivary gland, BM: bone marrow, SM: skeletal muscle, A-liver: adult liver, F-liver: fetal liver, SI: small intestine. Y axis indicates % ACTB, and error bars represent standard deviation of technical duplicates.
(TIF)

S1 Table The expression levels of known CEC markers in the RNA-seq data and the FANTOM5 database. (DOC)

S2 Table List of FANTOM consortium members. (DOCX)
