# Correction: Discovery of Molecular Markers to Discriminate Corneal Endothelial Cells in the Human Body

**DOI:** 10.1371/journal.pone.0129412

**Published:** 2015-05-26

**Authors:** Masahito Yoshihara, Hiroko Ohmiya, Susumu Hara, Satoshi Kawasaki, FANTOM consortium, Yoshihide Hayashizaki, Masayoshi Itoh, Hideya Kawaji, Motokazu Tsujikawa, Kohji Nishida

There is an error in the legend for Fig 2. Please see the complete, correct Fig 2 legend here.

**Fig 2:**
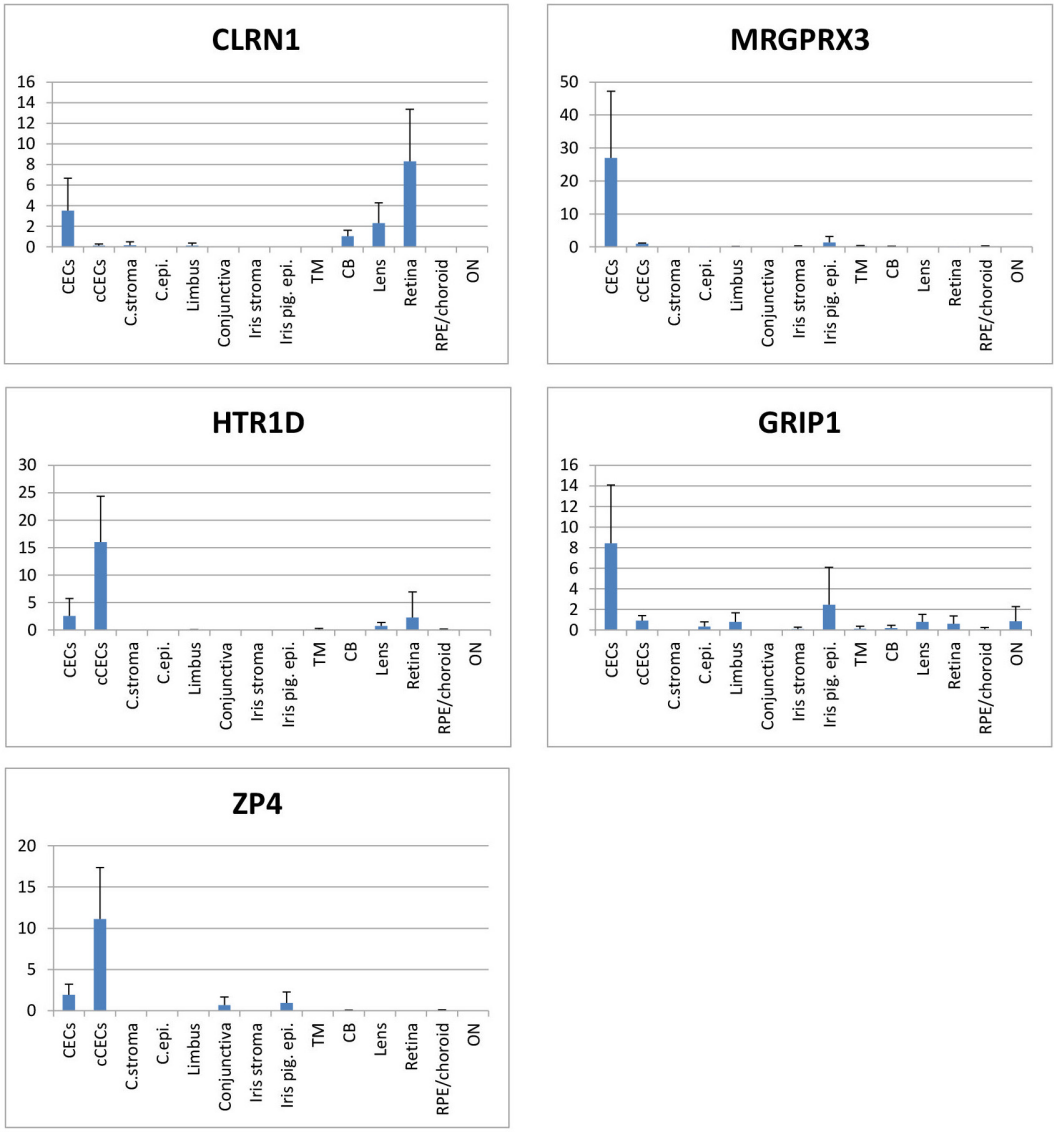
qRT-PCR analysis of 5 corneal endothelial cell marker candidate genes within ocular tissues. CECs: corneal endothelial cells, cCECs: cultured corneal endothelial cells, C.stroma: corneal stroma, C.epi.: corneal epithelial cells, iris pig. epi.: iris pigment epithelial cells, TM: trabecular meshwork, CB: ciliary body, RPE: Retinal pigment epithelial cells, ON: optic nerve. Y axis indicates % ACTB, and error bars represent standard deviation of four replicates.
