# Supplementary figures and images for: Discovery of Molecular Markers to Discriminate Corneal Endothelial Cells in the Human Body
Source: PLoS One. 2015 Mar 25;10(3):e0117581. doi: 10.1371/journal.pone.0117581 (PMC4373821; doi:10.1371/journal.pone.0117581)

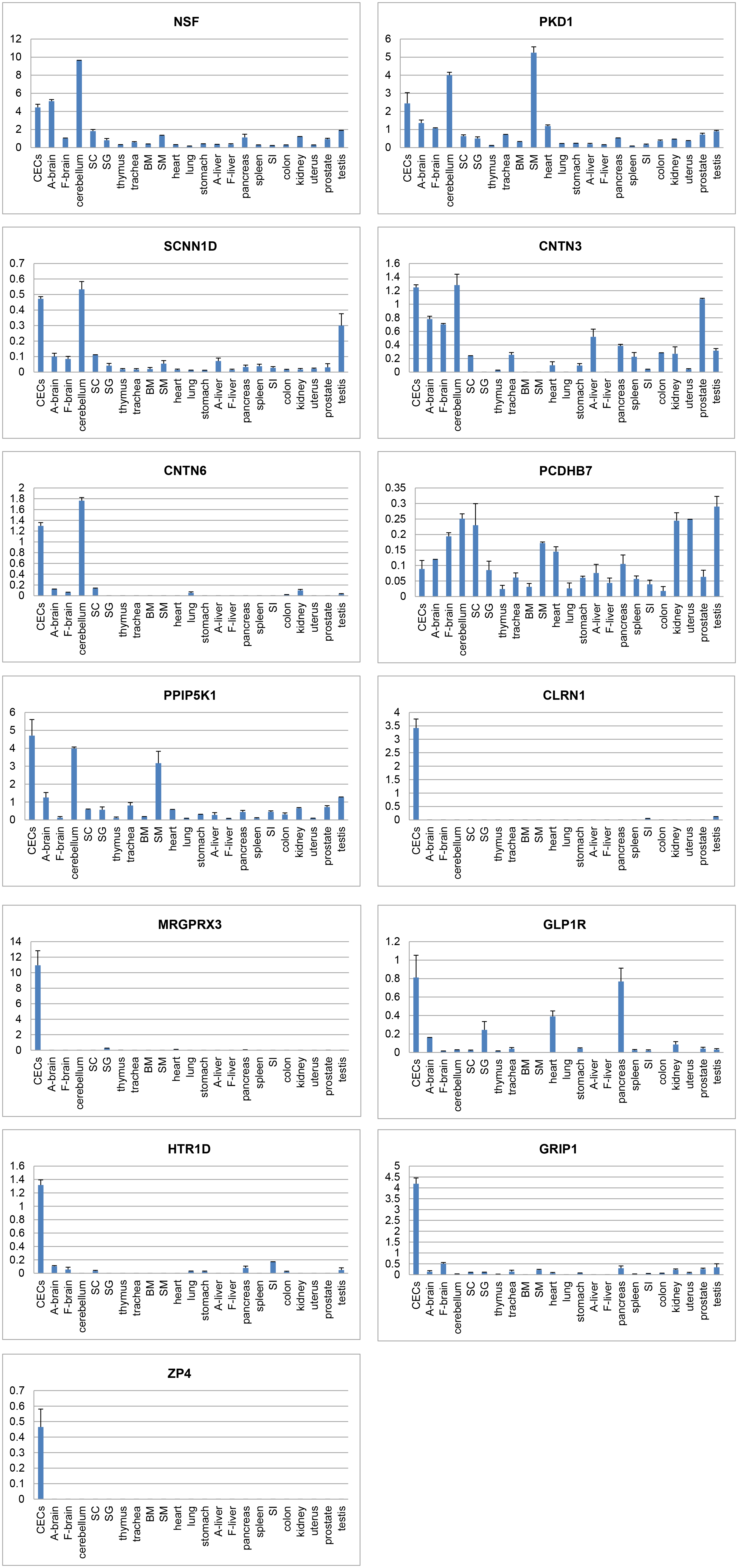

Supplement: S1 Fig — CECs: corneal endothelial cells, A-brain: adult brain, F-brain: fetal brain, SC: spinal cord, SG: salivary gland, BM: bone marrow, SM: skeletal muscle, A-liver: adult liver, F-liver: fetal liver, SI: small intestine. Y axis indicates % ACTB, and error bars represent standard deviation of technical duplicates. (TIF) [file pone.0117581.s001.tif]
