# Supplementary material for: Discovery of Molecular Markers to Discriminate Corneal Endothelial Cells in the Human Body
Source: PLoS One. 2015 Mar 25;10(3):e0117581. doi: 10.1371/journal.pone.0117581 (PMC4373821; doi:10.1371/journal.pone.0117581)
Supplement: S1 Table — (DOC) [file pone.0117581.s002.doc]

**Table S1. The expression levels of known CEC markers in the RNA-seq data and the FANTOM5 database.**

| **Gene** | **RNA-seq FPKM value** | | **FANTOM5 CAGE** | |
| --- | --- | --- | --- | --- |
|  | **adult CEC** | **fetal CEC** | **Primary sample**  **expresses highest (tpm)** | **Primary samples express >10tpm** |
| TJP1(ZO-1) | 54.02 | 44.56 | fetal heart(127.03) | 241 |
| ATP1A1 (Na^+^-K^+^-ATPase) | 105.29 | 67.32 | small intestine(1195.69) | 303 |
| CDH2(N-cadherin) | 57.87 | 32.05 | aortic smooth muscle cells (249.98) | 132 |
| SLC4A11 | 106.09 | 67.31 | thyroid (42.57) | 12 |
| COL8A2 | 71.97 | 92.22 | osteoblast(76.52) | 15 |
| CYYR1 | 51.02 | 10.41 | promyelocyte(108.18) | 70 |
| GPC4 | 44.80 | 73.29 | cerebral cortex astrocyte (355.81) | 158 |
| CD200 | 6.54 | 8.89 | mesothelial cells(311.11) | 179 |
